# Classifying Diverse Physical Activities Using “Smart Garments”

**DOI:** 10.3390/s19143133

**Published:** 2019-07-16

**Authors:** Mohammad Iman Mokhlespour Esfahani, Maury A. Nussbaum

**Affiliations:** 1 Department of Mechanical Engineering, The University of Michigan, Ann Arbor, MI 48105, USA; 2 Department of Industrial and Systems Engineering, Virginia Tech, Blacksburg, VA 24060, USA

**Keywords:** smart garment, smart textile system, wearable sensor, smart shirt, smart socks, physical activities, classification, human health

## Abstract

Physical activities can have important impacts on human health. For example, a physically active lifestyle, which is one of the most important goals for overall health promotion, can diminish the risk for a range of physical disorders, as well as reducing health-related expenditures. Thus, a long-term goal is to detect different physical activities, and an important initial step toward this goal is the ability to classify such activities. A recent and promising technology to discriminate among diverse physical activities is the smart textile system (STS), which is becoming increasingly accepted as a low-cost activity monitoring tool for health promotion. Accordingly, our primary aim was to assess the feasibility and accuracy of using a novel STS to classify physical activities. Eleven participants completed a lab-based experiment to evaluate the accuracy of an STS that featured a smart undershirt (SUS) and commercially available smart socks (SSs) in discriminating several basic postures (sitting, standing, and lying down), as well as diverse activities requiring participants to walk and run at different speeds. We trained three classification methods—K-nearest neighbor, linear discriminant analysis, and artificial neural network—using data from each smart garment separately and in combination. Overall classification performance (global accuracy) was ~98%, which suggests that the STS was effective for discriminating diverse physical activities. We conclude that, overall, smart garments represent a promising area of research and a potential alternative for discriminating a range of physical activities, which can have positive implications for health promotion.

## 1. Introduction

Physical activity refers to a range of human movements generated by the musculoskeletal system that result in energy consumption [1]. According to this definition, physical activity includes all static and dynamic postures in which an individual engages throughout the day. Physical activity is strongly linked to an increasing number of physical, emotional, and life-enhancing benefits. As such, the degree to which one engages in physical activity can impact one’s quality of life [2]. In contrast, a physically inactive lifestyle is directly tied to numerous adverse outcomes. For example, such as increased body mass index (BMI), musculoskeletal disorders, cardiovascular disease, Type 2 diabetes, high blood pressure, and obesity [2,3,4,5]. Conversely, physically active individuals tend to be at significantly lower risk for such diseases, and a review of several studies confirmed that the risks of cardiovascular disease and other causes of mortality are decreased by 35% and 33%, respectively, for physically active individuals [6]. In addition to the risks to one’s physical wellbeing, physical inactivity is also linked to mental disorders such as stress, anxiety, depression, low self-esteem, and reduced cognitive functioning [2,7]. There is also a well-documented monetary component associated with physical inactivity. For example, a recent study indicated that the annual cost of physical inactivity in the U.S., as measured in healthcare-related expenditures, was ~$117 billion between 2006 and 2011 [8]. In short, physical activity has several important impacts on human health, and a physically active life style is correlated with improvements in both physiological and psychological health [9].

To offset the risk for the range of disorders noted above, and to promote improved physical and emotional wellbeing, it is useful to recognize static postures and to quantify sedentary periods during a day. For this purpose, it is necessary to accurately determine the type of major daily activities in which a person engages, so that then it would be possible to identify static postures among those activities, and thereby help an individual to change their behavior from an inactive lifestyle to a more active one. To accomplish this, activity monitoring represents an increasingly effective way for determining an individual’s physical activity level during any given day. Indeed, many scholarly and commercially driven efforts are seeking to develop an accurate and reliable activity monitor that is applicable for diverse activity domains. To date, these monitors can be broadly categorized into subjective and objective types.

Subjective methods are typically used to assess physical activity through self-evaluations, with the two main strategies involving questionnaires and diaries [10]. These subjective methods are, overall, relatively low-cost, convenient, and applicable for a large population. Current subjective methods, however, like all self-reported data, share some limitations when used to discriminate physical activities—principally their low validity [10,11] and low reliability [11]. They also tend to be time-consuming and are at risk for biased reporting [12]. Finally, ensuring that they are culturally specific is an increasingly important component of such instruments [10].

Objective methods that rely on a range of underlying technologies have also been used to measure human behaviors and activities. Objective assessment systems currently available can be broadly categorized into two types: non-wearable and wearable systems [13]. The former type (e.g., video motion capture system) has been used mainly in laboratory environments, since sophisticated hardware or software is often needed. Wearable systems, though, are both portable and usable for diverse indoor and outdoor applications for ambulatory motion analysis; such applications include medical service systems (e.g., detection of falls or health problems), rehabilitation, ergonomics, and sport biomechanics [14,15,16,17,18,19,20,21,22,23,24,25,26]. Individuals being assessed for physical activity tend to prefer small, lightweight wearable devices that are easily operated and maintained, while also being compatible with daily activities [27,28,29]. Furthermore, an increased focus on wellness, coupled with advances in sensing technologies, has resulted in a sizable market for portable wearable systems compared to non-wearable systems; indeed, the market for wearable systems is predicted to grow to over $12 billion by 2022 [30]. This earnings potential has, in part, spurred researchers to develop and utilize wearable, non-invasive, low-cost, and lightweight devices for classifying a range of common activities [27,28,31,32,33]. For example, a recent review described the use of accelerometry-based devices placed on different body segments to discriminate physical activities [34]. It indicated that the use of accelerometry-based devices provides classification accuracy ranging from 85% to 95% [34]. Similarly, Karantonis et al. [35] used an accelerometer on the waist for detecting sitting, standing, walking, lying, and falls, and reported 91% accuracy using a decision tree method. By combining an accelerometer, gyroscope, and barometer, investigators reported being able to discriminate physical activities using the K-nearest neighbor (K-NN) classification method with 95% accuracy [36]. Massé et al. [37] utilized a combination of an inertial measurement unit and a barometric pressure sensor placed on the trunk to detect sitting, standing, walking, and lying, which yields an accuracy of ~90%. An instrumented shoe equipped with special insoles and an inertial measurement unit (IMU) recognizes a number of human movements (sit-to-stand, locomotion, and walking type) using the decision tree method, with an overall accuracy of 97% [38]. However, while they appear to be a promising solution in several applications [39,40], these wearable sensors are external devices that need to be attached on the body, and therefore they may change the appearance of garment and compromise usability [41].

An alternative and promising wearable technology is being used increasingly by physical activity researchers, namely interactive or smart textiles that have sensing material incorporated within them. So-called smart garments (SGs) [42,43] are becoming an important technology for diverse applications, such as in healthcare, the military, the consumer fitness realm, and gaming/sports [42,43,44]. This technology presents some significant advantages. For example, SGs can be easily tailored for individual usage; they are relatively inexpensive (potentially even disposable); and they can be implemented in close proximity to the body and are thus able to accurately capture physical movements [45]. For these reasons, SGs have received increasing interest in recent years for discriminating a range of activities. However, limited empirical evidence exists to support the implementation of SGs for accurately discriminating physical activities.

Based on this lack of experimental evidence, an exploratory study was designed to assess the feasibility and accuracy of using a specific smart textile system (STS) to classify several basic physical activities. Thus, we completed a study to evaluate the ability of two SGs for classifying diverse physical activities: (1) a commercially available pair of smart socks (SSs) that rely on textile pressure sensors; and (2) a customized smart undershirt (SUS) that utilizes textile strain sensors. Additional objectives of this study were to explore the relative merits of these two SGs, both separately and in combination, and to compare the relative performance of several common classification methods. Furthermore, we sought to identify the most effective subset of sensors for the SUS, based on accuracy in classifying several basic physical activities. For these purposes, a lab-based experiment was undertaken to determine how accurately an STS can classify several basic postures (sitting, standing, and lying down) and diverse physical activity types (e.g., walking and running at different speeds). By discriminating diverse physical activities using SGs, we hope to more accurately and efficiently detect these activities, and determine the time spent in different active tasks. From doing so, both healthcare providers and individuals could have the information they need to promote health and wellbeing, for example to quantify sedentary periods and modify a sedentary lifestyle to a more active one.

## 2. Materials and Methods

### 2.1. Participants

A total of 11 participants from the local student population and local community completed the study (Table 1). Three inclusion criteria were confirmed for each participant: (a) they needed to be moderately physically active; (b) they could not have experienced any current or recent history of musculoskeletal disorders; and (c) they needed to be able to wear the SSs and fit comfortably into the single SUS that was available. Experimental procedures were approved by the Virginia Tech Institutional Review Board, and participants completed an informed consent procedure approved prior to beginning the study.

### 2.2. Experimental Procedures

An experiment was designed to investigate the efficacy of utilizing an STS (including both SSs and the SUS) in discriminating basic human postures and common physical activities types. Prior to the actual data collection sessions, all participants completed a training session, during which they were first sized for the SSs (from medium, large, and X-large sizes) and specified shoes (Jogger style, Athletic Works Shoe). They were then asked to walk and run on a treadmill (SOLE F63, SOLE Fitness, Salt Lake City, UT, USA) while wearing the STS, during which we obtained their preferred walking and running speeds [46].

In the experimental session, participants were asked to complete 11 basic physical activities: standing to lying down & reverse (A1), lying down (prone) (A2), standing on both feet (A3), standing-to-sitting & reverse (A4), sitting on a chair (A5), slow walking (A6), comfortable walking (A7), fast walking (A8), comfortable running (A9), fast running (A10), and stairs climbing up/down (A11). Activities A5–A10 were performed on the noted treadmill. The three walking and two running speeds were set according to each participant’s preferred walking and running speeds, (specifically slow, comfortable, and fast indicated 80%, 100%, and 120% of their preferred speed, respectively) [46]. Table 2 provides results regarding the three different walking speeds (slow, comfortable, and fast) and the two running speeds (comfortable and fast).

The order of the 11 activities was assigned to participants using a partially balanced Latin square [47] to minimize the potential for order-related confounding effects. Each of the transition activities (i.e., A1 and A4) was repeated 10 times, and each of these activities lasted ~2 s. The remaining activities were done repeatedly for at least 120 s each, in order to collect sufficient samples for subsequent classification model developing and testing, as described below [48]. Moreover, we encouraged the participants to utilize different strategies to perform the activities each time, to include more variability for the purpose of enhanced generalization of results. To normalize signal magnitudes from the SSs and SUS and thereby facilitate activity classification, we asked the participants to adopt two reference postures [49]: (1) sitting on a chair and lifting both feet (for normalizing the signals from the SSs); and (2) standing upright on both feet with the trunk erect and the arms hanging relaxed (for normalizing signals from the SUS).

### 2.3. Smart Textile System

**Figure 1:**
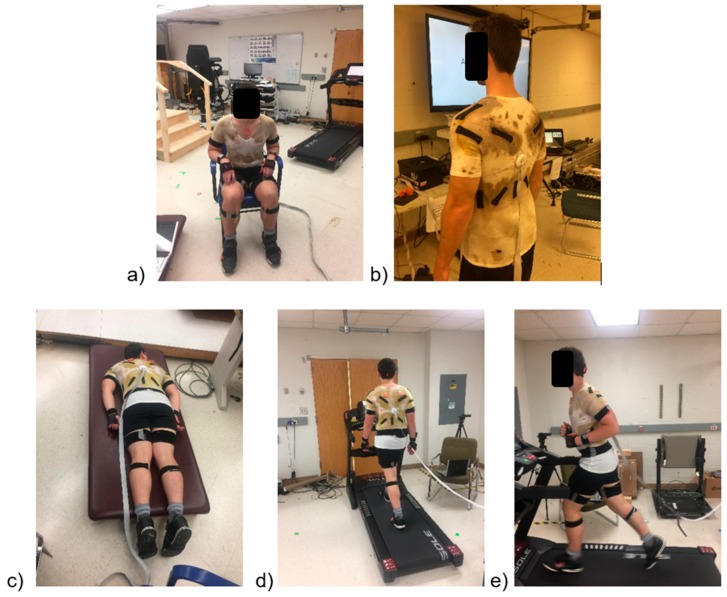
Illustrations of a participant wearing two smart garments (smart shirt and smart socks) while: (**a**) A5: sitting, (**b**) A3: standing, (**c**) A2: lying down, (**d**) A6: walking, and (**e**) A9: running.

All participants donned the two SGs (SSs and SUS), which were included as representatives of activity monitors for the lower and upper extremities, respectively; together they formed this study’s STS. The commercially available Sensoria SSs (Sensoria Inc., Redmond, WA, USA, www.sensoriafitness.com) featured three textile pressure sensors integrated into the heel, the first metatarsal bone, and the fifth metatarsal bone. SSs that employ integrated pressure textile sensors can measure foot pressure relatively easily. These SSs can be used either within a pair of shoes for mostly outdoor applications (e.g., running) or by themselves for indoor or more outdoor applications [50]. An important component of the Sensoria socks is that they include two wireless transmitters as anklets, providing a 32 Hz sampling rate; thus, the socks feature limited wiring and did not constrain normal activities or reduce the wearer’s comfort. In contrast, the SUS, which was developed and calibrated in our lab as described earlier [49], included 11 stretchable textile sensors [51] placed on the low-back and shoulder regions to record thorax vs. pelvis and 3D shoulder motions, respectively. These sensors were developed by coating electroactive polymers (i.e., polymerization) on a fabric using manual screen printing, and they quantify strain in the fabric by measuring resistance changes between the two ends of each sensor [51]. The sampling rate for the SUS was 1000 Hz. We purposefully selected an undershirt, in accordance with a recent study indicating that participants preferred a short-sleeved T-shirt over other types of SGs [41]. Furthermore, the upper body typically involves a larger range of activity, as well as variability, compared to the lower body, and was thus thought to be more effective in classifying physical activities. Figure 1 depicts a participant wearing both SGs during several of the physical activities investigated.

A 4th-order bi-direction Butterworth filter was used to filter raw data from the SSs and SUS, with a cutoff frequency of 5 Hz for both [52]. We then resampled all data at 20 Hz, which represents an appropriate sampling rate for physical activities [48,53]. These preprocessing methods were implemented using MATLAB (2016, The MathWorks, Inc., Natick, MA, USA).

### 2.4. Activity Classification

As noted above, it is important to classify different types of physical activities. Here, the specific problem was to classify the 11 simulated physical activities through the use of SGs. It is unrealistic to presume (at least initially) that a single, optimal classification method can be derived for all conditions of interest [54,55]. For this reason, researchers have typically utilized and compared several classification methods to achieve better accuracy [54,55,56,57,58]. Similarly, we implemented three relatively common classification methods—K-NN, linear discriminant analysis (LDA), and artificial neural network (ANN)—each implemented using MATLAB (2016, The MathWorks, Inc., Natick, MA, USA).

We developed a number of different types of classification models, differing in: (1) whether they were at the individual or group levels; (2) whether the inputs included data from the SSs, the SUS, or their combination (STS); and (3) the classification method employed (K-NN, LDA, or ANN). At the individual level, we adjusted the inputs for these methods to form the raw signals for the SSs (six features) and SUS (11 features), and the output target from the relevant activity patterns (A1–A11) for each individual participant (i.e., individual level). However, we used all data from all participants as the inputs for the classification methods at the group level. We randomly selected data from all participants for training the group-level models. We considered these two types since we wanted to compare the accuracy of SGs in both potential applications. In other words, we sought to determine whether a system might need to use a personalized classification model for each individual or whether it was feasible to use a general model developed based on the data from multiple individuals. We also, as noted, trained the different classification models using data from each SG separately, as well as in combination. Therefore, we developed nine classification models at the group level (3 input sets × 3 classification methods), and 99 classification models at the individual level (11 participants × 3 inputs sets × 3 classification methods). A 5-fold cross-validation technique was used to avoid overfitting of the K-NN and LDA models. For ANN models, the complete dataset was randomly divided into training (70%) and testing (30%) subsets according to conventional methods [59]. We set k to be 10 for the K-NN method after several initial iterations [54].

We then evaluated the performance of these classification models using common metrics, global accuracy, sensitivity (recall), specificity, precision, accuracy, and the F-score [54], which were determined using the testing data subset. Furthermore, we created confusion matrices based on results obtained from the entire set of data (both training and testing subsets); these were used to assess the performance of the classification models for each activity and to identify the most confused specific pairs among the set of 11 activities. Using the entire dataset for confusion matrices provided a more complete assessment of misclassified activities, especially for identifying the most confused activities pairs. Additionally, the confusion matrix provided the percentages of precision, false discovery rate, sensitivity, and false negative rate for each activity.

### 2.5. Most Effective Sensors in the SUS

We used the estimated Bayes accuracy method to determine the most effective subsets of the 11 SUS sensors, based on the accuracy in classifying physical activities. The best possible accuracy for any classification problem is known as Bayes [60] which is independent of any specific classification methods and depends only on the distribution of the classes. Noshad and Hero [61] proposed a method to compute a tight bound on the Bayes accuracy. We used their method here to find the most effective subset of SUS sensors. This method also helped to determine the best possible accuracy that can be achieved by the SUS. In our analyses, we chose increasing subsets. We first chose a subset of size one, and thereby determined which sensor could classify the activities with the best accuracy using a single sensor. This was performed by simply comparing the Bayes accuracies among all single sensors. Next, we added another sensor in each step and identified the set of sensors that provided the best classification accuracy. We continued this procedure until we included all 11 sensors. The advantage of this sensor selection method is that it does not depend on any specific classification method (i.e., it is generic).

## 3. Results

Table 3 provides a summary of global accuracy for each classification model at both the group and individual levels. At the group level, global accuracies using the K-NN, LDA, and ANN methods were in the range of ~97–98%, ~15–47%, and ~90–98%, respectively. Models developed using LDA demonstrated relatively poor classification performance at the group level when using the SSs, SUS, and STS, while the other two methods (K-NN and ANN) yielded comparable global accuracies with all three systems. Although global accuracies with the STS using both K-NN and ANN classification methods were the same (98%), global accuracies for SSs and SUS using the K-NN method were slightly better than when using ANNs. Note that using SUS data resulted in improved global accuracy relative to the SSs for all classification methods except K-NN, for which the results were nearly similar. In general, the global accuracy at the individual level was higher than at the group level.

F-scores are provided in Table 4 for all classification models at the group level. Similar to the results for global accuracy, F-scores resulting from the LDA method were relatively lower. The K-NN approach yielded the best performance, with F-scores for all activities exceeding 0.90. Activities A2, A3, and A5 had the best classification performance (i.e., 0.99) for both SSs and SUS using the K-NN method.

Table 5 shows the remaining classification performance metrics for models developed using the K-NN method at the group level for each activity. Note that we only presented confusion matrices for the K-NN method, since models developed using this method provided the best performance based on global accuracy and F-score. Each of these metrics was >0.9 for all activities (maximal values for these metrics = 1.0).

**Figure 2:**
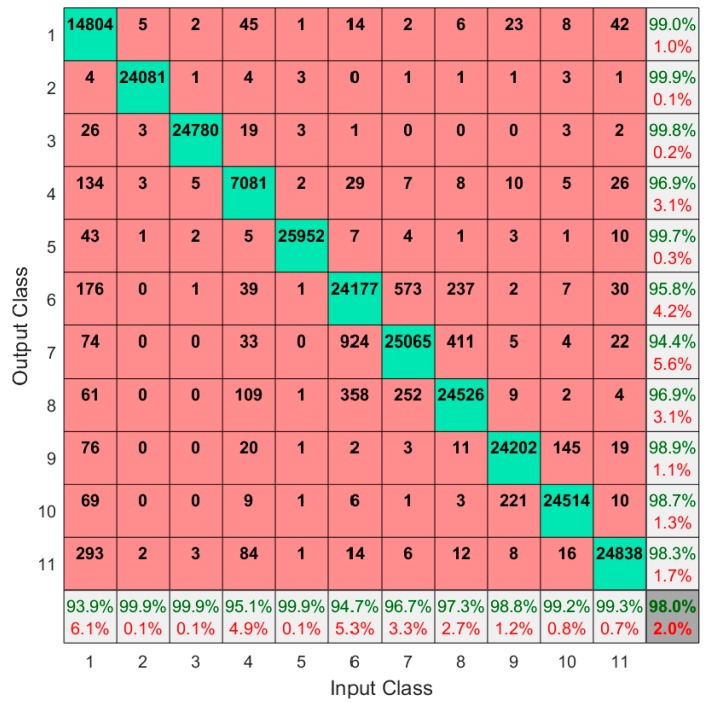
Confusion matrix when using the K-NN method at the group level (i.e., all participants), using input from both SSs and SUS (i.e., the complete STS). Input and output classes correspond to the 11 simulated physical activities. Cells on the main diagonal (green color) and off-diagonal (red color) indicate the numbers of correctly and incorrectly classified observations of each activity, respectively. Cells in the right-hand column provide the percentages of precision (green font) and false discovery rate (red font) for each activity. Cells in the lowest row provide the percentages of both sensitivity (green font) and false negative rate (red font) for each activity. The cell at the bottom-right corner (gray color) provides global accuracy.

Figure 2 provides confusion matrices for models developed using the K-NN method at the group level. Note that we only presented confusion matrices for the K-NN method, since models developed using this method provided the best performance based on global accuracy and F-score. We had 11 participants, each of whom performed 11 simulated physical activities. As noted earlier, each of the transition activities (i.e., A1 and A4) was repeated 10 times, while the remaining activities were done repeatedly for at least 120 s. The different activities required different durations. At our re-sampled rate of 20 Hz, this means there were ~650–2200 samples per participant, and ~7100–25,000 total samples for each activity (for example, Figure 2 shows 14,804 total samples for A1). The most confused pairs of physical activities using the SSs, SUS, and STS were A6 (slow walking) and A7 (comfortable walking). A7 (comfortable walking) and A8 (fast walking) were the second most confused activities.

**Figure 3:**
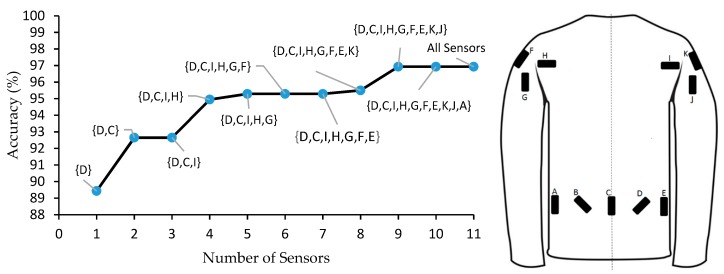
Results of applying the Bayes accuracy method (left) to identify effective subsets of sensors, from the total of 11 sensors in the SUS (sensors are labeled in the figure on the right, using letters A-K).

Figure 3 shows the results of using the Bayes accuracy method to find the most effective subsets of SUS sensors on classifying physical activities. Sensor D was selected as the most effective single sensor, with an accuracy of ~89%. Adding sensor C could increase accuracy by roughly 4%. After adding two more sensors on the shoulder area, the accuracy was further increased by ~2%. Finally, the best accuracy could be achieved using nine sensors (97%); adding sensors A and B did not improve accuracy.

## 4. Discussion

Our goal for this study was to assess the ability of textile-based sensor systems to help classify different physical activities. This is the first study to evaluate SSs and SUS separately and their combination in classifying physical activities, to our knowledge. For this purpose, we completed an experiment to evaluate the accuracy of a specific STS featuring two SGs (SSs and a smart shirt), using three classification methods in classifying diverse types of simulated physical activities. Classification results indicated that the STS could discriminate among the several physical activities, with accuracy levels of 99% at the individual level and ~98% at the group level (Table 3). Of the three classification approaches tested, the K-NN method provided the best classification performance based on global accuracy and F-scores (Table 3 and Table 4). No clear differences in performance were evident between using inputs from the SSs and SUS with the K-NN method. However, classification performance was better with data from the SUS and the SSs when using the LDA and ANN methods. The reason for the latter differences in performance may be that most of the physical activities simulated here involved more variability in upper vs. lower extremity behaviors. For example, there may be important differences in shoulder motions while walking and running, with relatively less differences in pressure patterns at the feet. While using data from the STS yielded slightly better classification performance than when using either of the two components separately, the improvement in classification accuracy was only modest (1–2%). It thus appears that the use of either SSs or the SUS may be sufficient, with no major benefit obtained from using both of these wearable systems. Of note, using data from either the SSs or SUS was also shown earlier to be effective at classifying diverse occupational tasks [25]. We also found that global accuracies using data from the SSs and SUS were similar for models developed at the individual and group levels (Table 3). Thus, it may be feasible to develop and implement a general model for multiple users.

Accuracy of the current STS was found to be at least comparable to the earlier studies using different wearable activity monitors. It should also be noted that participants may prefer using SGs rather than systems based on inertial measurement units [41]. There are several possible explanations for this high accuracy in identifying diverse physical activities. First, we printed the sensors in the SUS at specific locations to capture upper body movements [49]. Second, the pressure sensors on the bottom of SSs were also placed at three positions to capture movements of the feet. Third, six of activities investigated here (A1–A4, A10, and A11) involved very different ranges of movements.

Here, slow walking (A6) and comfortable walking (A7) were the most confused pair of activities among the 11 physical activities simulated (Figure 2). This misclassification may have resulted from the obvious kinematic similarity between these two activities. Additionally, the slow walking speed was set relative to each participant’s preferred speed (i.e., 80%). Thus, there may have been insufficient differences in kinematics between the slow and comfortable walking speeds to allow for more accurate discrimination (Table 2). Indeed, this overlap may also account for the second most confused pair of activities: comfortable walking and fast walking. Overall, the most frequent misclassifications were between activities requiring different speeds of walking or running (Figure 2). These misclassifications may also have occurred as a result of overall classification approach used here. For example, we did not extract any temporal features. Thus, future work is needed to investigate the merits of including temporal features and/or feature selection methods in discriminating between different speeds of walking and running using SGs.

Based on the Bayes accuracy method, the best possible accuracy that can be achieved using the SUS is on the order of 97%. Using K-NN methods could reach an accuracy of 96% with the SUS at the group level. Thus, we can conclude the K-NN is almost the best classification method for the SUS. From examining effects sensor subsets using the same Bayes method, we found that the best accuracy could be achieved with nine of the initial 11 SUS sensors. Specifically, two can be removed without loss of classification performance. If a slight decrease in performance is acceptable, as few as 2–4 sensors may be sufficient (Figure 2).

Although the current results support the feasibility of using an STS for classifying physical activities, some limitations in the study must be addressed. First, we included a relatively small sample size of only healthy, young volunteers, which precludes the possibility of generalizing the results of this study to a population consisting of older adults and/or individuals with medical conditions [62]. Second, this study required participants to engage in simulated basic physical activities. Therefore, the extent to which results of this study can be generalized to a wider set of activities is unknown [11,63]. Furthermore, simulated activities may involve different behaviors compared to those in real life [64]. In fact, a number of diverse activities do occur during a person’s typical day that go beyond sitting, standing, walking, and running. Third, the sampling rate use here was ~20 Hz, which prior research has indicated may be sufficient for physical activities [48,53]. However, this sampling rate may be insufficient for accurately classifying highly dynamic activities. Thus, the findings detailed herein cannot be extrapolated to high-speed activities, such as during certain sports. Fourth, our SUS transferred signals from the shirt to an electronic board via wires, and these would likely restrict the performance of physical activities in real-life settings. Future efforts are needed to modify such garments, such as with wireless transmitters that would not impede normal activities. Additionally, the SUS may be improved for the shoulders, since it had weaker performance in terms of angle estimation than in the lumbar area [49], and, as noted earlier, participants had to “fit” to the single SUS that was available. For future applications, improving accuracy at the shoulder may, in turn, enhance the ability of the SUS in classifying physical activities, and diverse sizes of the SUS will need to be developed. Fifth, we did not employ any automated data segmentation or feature extraction. In practice, a continuous data stream from STS sensors will need to be divided into smaller subsets, although here segmentation was done manually (start to end of a given activity). While feature extraction could enhance classification performance, a featureless approach was used here, which decreased computation cost. The fairly good classification performance we found may also suggest that feature extraction may have limited additional value. Another limitation related to using data streams is potential correlations between samples that might result in inflated classification accuracy for our SGs. To address this, secondary analyses were completed using data segmentation (temporal windows, size = 1 s), from which we extracted the following features: mean, median, standard deviation, maximum, and minimum. Using temporal windows with these features increased classification accuracy by 1%. Furthermore, the Bayes accuracy method showed that the best accuracy that could be achieved using the SUS was 97%. Thus, we concluded that obtaining high accuracy when classifying physical activities using SGs is feasible, and that using data stream seems not critical here since slightly better accuracy could be obtained with temporal windows. However, additional work is suggested to both address data segmentation and assess the value of extracting features related to diverse physical activities.

## 5. Conclusions

In this study, we evaluated the potential for using two SGs including SSs and SUS—as part of a system—to classify diverse physical activities. Data obtained from SSs, the SUS, and the combined system (STS) could effectively discriminate between several diverse physical activities with fairly high levels of accuracy and using standard classification methods. Our results also suggested that the use of single classification model, developed using data from multiple individuals, could be effective when applied across individuals. Based on our findings, we hope to facilitate future work that more effectively discriminates additional activity types that may help or hinder health and fitness activities. Such information will likely be of use to both healthcare practitioners and individuals. More specifically, results from future investigations could provide strategies for helping to accurately identify injury risk factors associated with human movement. For example, an STS may be useful in quantifying sedentary periods with the goal of modifying a largely sedentary lifestyle to a more active one. Indeed, mounting evidence shows that a sedentary lifestyle is deleterious to human health and emotional wellbeing across a range of factors, such as increased BMI [65], the effectiveness of breathing [66], a higher risk for developing cancer [65] and cardiovascular disease [67] and diabetes [65,68]. Therefore, an accurate and reliable wearable system, such as an STS for activity classification, could be useful for healthcare providers to quantify sedentary periods, with the goal of devising strategies for changing an inactive lifestyle to a more active one. Another potential future application is to classify physical activities among older individuals and those with diverse health problems, since these systems may be useful for monitoring health status.

## 6. Patents

A U.S. patent has been filed for the SUS; disclosure # 62/641,448.

## Figures and Tables

**Table 1:** Summary of participant characteristics (SD = standard deviation).

Measure	Mean (SD)	Range
Age (years)	21.3 (2.5)	18–26
Body mass (kg)	76.2 (8.2)	64.4–86
Stature (cm)	174.5 (7.4)	163–186
BMI (kg/m^2^)	25.0 (2.6)	22.4–29.4

**Table 2:** Walking and running speeds used in the experiment for each participant (PX is an abbreviation for participant number, units are km/h, and SD is an abbreviation for standard deviation).

		P1	P2	P3	P4	P5	P6	P7	P8	P9	P10	P11	Mean (SD)
Walking	Slow	2.1	1.9	2.0	2.1	2.1	2.1	2.0	2.2	1.9	2.2	2.3	2.08 (0.12)
	Comfortable	2.6	2.4	2.5	2.6	2.6	2.6	2.5	2.8	2.4	2.7	2.9	2.6 (0.15)
	Fast	3.1	2.9	3.0	3.1	3.1	3.1	3.0	3.4	2.9	3.2	3.5	3.12 (0.19)
Running	Comfortable	5.6	4.6	5.7	5.5	5.5	5.8	6.0	4.2	5.4	6.3	5.1	5.43 (0.6)
	Fast	6.7	5.5	6.8	6.6	6.6	7.0	7.2	5.0	6.5	7.6	6.1	6.51 (0.74)

**Table 3:** Global accuracy (percentage) for each classification method at the individual and group levels for the smart garments (SSs), smart undershirt (SUS), and smart textile system (STS).

Model		Individual Level											Group Level
		P1	P2	P3	P4	P5	P6	P7	P8	P9	P10	P11	
K-NN	SSs	97	97	98	99	98	99	96	98	97	96	96	97
	SUS	95	97	97	98	98	97	96	95	98	96	91	96
	STS	97	99	99	99	99	99	98	98	99	98	96	98
LDA	SSs	69	89	90	91	83	92	72	81	90	75	83	15
	SUS	87	91	89	92	96	94	88	84	96	90	82	42
	STS	94	96	97	97	99	97	92	96	98	96	93	47
ANN	SSs	95	94	97	98	98	98	93	93	95	90	93	90
	SUS	95	99	98	99	98	98	97	95	98	88	90	94
	STS	97	99	99	99	99	99	98	99	99	98	98	98

K-NN: k = 10 in k-nearest neighbor, LDA: linear discriminant analysis, ANN: artificial neural network. P1–P11 are participant numbers.

**Table 4:** F-scores obtained for each activity using different classification models at the group level.

Model		A1	A2	A3	A4	A5	A6	A7	A8	A9	A10	A11
K-NN	SSs	0.96	0.99	0.99	0.97	0.99	0.94	0.93	0.95	0.98	0.99	0.97
	SUS	0.92	0.99	0.99	0.9	0.99	0.93	0.93	0.95	0.96	0.96	0.97
	STS	0.96	0.99	0.99	0.95	0.99	0.95	0.95	0.97	0.98	0.98	0.98
LDA	SSs	0	0.2	0.21	0	0.13	0.17	0.09	0.08	0.19	0.13	0.11
	SUS	0.26	0.78	0.52	0.02	0.52	0.23	0.26	0.38	0.39	0.5	0.23
	STS	0.79	0.6	0.11	0.58	0.38	0.31	0.43	0.45	0.49	0.3	0.35
ANN	SSs	0.78	0.97	0.92	0.84	0.97	0.82	0.83	0.84	0.96	0.96	0.88
	SUS	0.88	0.99	0.99	0.81	0.99	0.90	0.90	0.92	0.94	0.94	0.95
	STS	0.97	0.99	0.99	0.97	0.99	0.96	0.96	0.97	0.99	0.99	0.98

A1–A11 are activities.

**Table 5:** Classification performance for each activity (A1–A11) using K-NN models at the group level.

	Sensitivity			Specificity			Precision			Accuracy		
	SS	SUS	CSTS	SS	SUS	CSTS	SS	SUS	CSTS	SS	SUS	CSTS
A1	0.96	0.87	0.93	0.99	0.99	0.99	0.95	0.97	0.99	0.99	0.99	0.99
A2	0.99	0.99	0.99	0.99	0.99	0.99	0.99	0.99	0.99	0.99	0.99	0.99
A3	0.99	0.99	0.99	0.99	0.99	0.99	0.99	0.99	0.99	0.99	0.99	0.99
A4	0.98	0.86	0.95	0.99	0.99	0.99	0.96	0.94	0.96	0.99	0.99	0.99
A5	0.99	0.99	0.99	0.99	0.99	0.99	0.99	0.98	0.99	0.99	0.99	0.99
A6	0.93	0.92	0.94	0.99	0.99	0.99	0.96	0.93	0.95	0.98	0.98	0.99
A7	0.94	0.95	0.96	0.99	0.98	0.99	0.92	0.91	0.94	0.98	0.98	0.99
A8	0.94	0.95	0.97	0.99	0.99	0.99	0.95	0.94	0.96	0.99	0.99	0.99
A9	0.99	0.96	0.98	0.99	0.99	0.99	0.98	0.97	0.98	0.99	0.99	0.99
A10	0.99	0.96	0.99	0.99	0.99	0.99	0.99	0.95	0.98	0.99	0.99	0.99
A11	0.96	0.98	0.99	0.99	0.99	0.99	0.98	0.96	0.98	0.99	0.99	0.99
Mean	0.97	0.95	0.97	0.99	0.99	0.99	0.97	0.96	0.98	0.99	0.99	0.99
